# Household Food Insecurity Narrows the Sex Gap in Five Adverse Mental Health Outcomes among Canadian Adults

**DOI:** 10.3390/ijerph16030319

**Published:** 2019-01-24

**Authors:** Geneviève Jessiman-Perreault, Lynn McIntyre

**Affiliations:** Department of Community Health Sciences, Cumming School of Medicine, University of Calgary, Calgary, AB T2N 1N4, Canada; gjessima@ucalgary.ca

**Keywords:** household food insecurity, mental health, sex, Canadian adults

## Abstract

The sex gap (i.e., the significant difference in an outcome between men and women) in the occurrence of a variety of mental health conditions has been well documented. Household food insecurity has also repeatedly been found to be associated with a variety of poor mental health outcomes. Although both sex and household food insecurity have received attention individually, rarely have they been examined together to explore whether or how these indicators of two social locations interact to impact common mental health outcomes. Using a pooled sample (N = 302,683) of the Canadian Community Health Survey (2005–2012), we test whether sex modifies the relationship between household food insecurity assessed by the Household Food Security Survey Module and five adverse mental health outcomes, controlling for confounding covariates. Although the sex gap was observed among food secure men versus women, males and females reporting any level of food insecurity were equally likely to report adverse mental health outcomes, compared with those reporting food security. Therefore, household food insecurity seems to narrow the sex gap on five adverse mental health outcomes.

## 1. Introduction

The sex gap (i.e., the significant difference in the occurrence of a health outcome between males and females) in mental health conditions has been consistently documented and re-examined [1,2,3]. The sex gap in mental health outcomes (typically cited as a nearly 2:1 ratio for women versus men reporting depression [4]) has also remained stable across decades. While the exact mechanism underlying drivers of the sex gap in mental health outcomes remains elusive, researchers have begun to examine the sex gap in the context of other important social determinants of health such as age [5], marital status [6], and sexual orientation [7]. This study examines the relationship between sex and five common mental health outcomes in the context of household food insecurity.

### 1.1. Household Food Insecurity in Canada and Adverse Mental Health Outcomes

Household food insecurity is operationally defined as the lack of access to food because of financial constraints [8] and in Canada, it is measured through national survey responses to the Household Food Security Survey Module (HFSSM) [9,10]. Using this metric, recent national estimates indicate that in 2012, 12.5% of Canadian households experienced some form of food insecurity (4.1% marginal food insecurity, 5.7% moderate food insecurity, and 2.7% severe food insecurity) [8]. Certain subsets of the population—groups most often associated with material deprivation—have a disproportionate risk of reporting household food insecurity; this includes Indigenous Canadians, African Canadians, households that rely on social assistance as their main income source, lone-mother led households, and those who rent rather than own their own home [8].

There is a large and robust body of literature establishing the relationship between household food insecurity and a variety of physical health problems. Adults and children living in food insecure households report poorer physical health; increased physical limitations; and higher prevalence of diabetes mellitus, heart disease, and other chronic conditions [11,12,13]. Furthermore, those living in food insecure households with pre-existing chronic health problems, such as diabetes, experience increased difficulties managing those conditions [14].

In addition to being associated with poorer physical health, there is a growing body of evidence that household food insecurity is associated with poor mental health and an increased risk of reporting certain mental health conditions, including psychological distress [15], mood and anxiety disorders [12,15], suicidal ideation [15], self-reported fair/poor mental health [16,17], depression [13,17,18,19], and psychiatric morbidity [20]. Recent research has reported that increasing severity of household food insecurity is associated with a graded increase in the risk of reporting six common mental health outcomes among Canadian adults [21]. 

Household food insecurity is hypothesized to be associated with poor mental health because of the unique social, physical, and psychological stresses associated with being in a food insecure household [22]. Interestingly, there is evidence that the relationship between household food insecurity and mental health could be bidirectional. Managing a food insecure household is extremely difficult and requires substantial planning [23]; therefore, individuals with pre-existing mental health conditions may be at increased risk of becoming food insecure as a result of the impact of the known symptoms of mental health conditions, such as a lack of energy, fatigue, loss of interest, and impairment of decision making, on the ability of the individual to manage a food insecure household [24,25].

### 1.2. Household Food Insecurity, Gender/Sex, and Mental Health 

Much of the research studying the impact of gender (i.e., the sociocultural expression of biological sex) on household food insecurity and mental health has been conducted on lone mothers. Researchers focusing on this topic have observed that a disproportionate number of food insecure households are led by mothers with a history of depression, psychosis spectrum disorder, or domestic violence [24]. Mothers reporting household food insecurity are also at increased risk of either a major depressive episode or a generalized anxiety disorder at every level of household food insecurity severity (21% for moderate, 30.3% for severe) compared with food secure mothers (16.9%) [19]. Food insecure women may occupy a distinct social position that makes them more susceptible to food management stressors. For example, women have been shown to protect other household members against food insecurity by reducing their food intake to allow other household members to have more food [26,27]. Moreover, women predominantly hold the responsibility for providing and preparing food, which, in the context of food insecurity, may increase levels of stress felt by women [27]. 

Comparatively little research has been conducted on the mental health of males reporting household food insecurity. The results from in-depth interviews indicate that food insecure men report similar precursors to mental illness as women, such as feelings of powerless, guilt, embarrassment, shame, inequity, and frustration [28]. These emotions, in conjunction with heightened levels of stress associated with food insecurity, could plausibly result in higher levels of mental health conditions. In cross-sectional surveys, men experiencing household food insecurity report a higher prevalence of mood or anxiety disorders compared with food secure men, but those figures are lower than the rates of mood and anxiety disorders observed in food insecure women [12]. Past research on the mental health of males has highlighted that simply being male may not provide equal privilege in mental health, particularly for males who occupy different social locations of disadvantage [29]. We suggest that household food insecurity may be one such social location of disadvantage for males. 

This study’s research questions are specifically as follows:

1. How is household food insecurity related to the reporting of five adverse mental health outcomes (depressive thoughts in the past month, anxiety disorders, mood disorders, suicidal ideation, and self-reported mental health) in Canadian adult men and women?

2. How is the sex gap in the reporting of five adverse mental health outcomes in Canadian adults changed by concurrent consideration of household food insecurity status, controlling for common socio-demographic covariates? 

## 2. Materials and Methods 

### 2.1. Data Source

The study sample (N = 302,683) was generated by pooling four cycles (Cycle 3.1 [2005], 2007–2008, 2009–2010, and 2011–2012) of the Canadian Community Health Survey (CCHS). The CCHS is a nationally representative series of cross-sectional surveys structured to collect information annually on a variety of issues relating to health including health status, health care utilization, and health determinants [30]. The target population, sampling procedure, and sample sizes are all determined by Statistics Canada. The CCHS is divided by health region and reflects estimates according to health region and province/territories, as well as the Canadian population as a whole. The CCHS collects data from a randomly selected person within a household aged 12 or older residing in a dwelling in the ten provinces and three territories. Individuals living on reserves or Crown land, in institutions, in remote regions, or who are members of the Armed Forces are not included in the survey. The CCHS data sample represents approximately 98% of the Canadian population aged 12 years or older [30]. It is important to note that the survey only captures biological sex, and not gender per se.

The CCHS questions are designed for computer-assisted interviewing with pre-programmed questions, content flow, and allowable responses (ranges or answers). Half of the interviews take place by telephone, while the other half take place in person. Participation in the CCHS is voluntary and responses are kept strictly confidential [30]. 

Given the difference in sample sizes between the four cycles, the existing survey weights (determined by Statistics Canada) were adjusted depending on their contribution to their total pooled sample sized. Once the individual cycles’ sample weights were adjusted, the cycles were combined and the pooled dataset was treated as one sample from a single population with a sample size of N = 515,421 prior to exclusions. 

### 2.2. Exclusion Criteria

The population of interest in this study is working-age Canadian adults, aged 18–64 years, living in the ten provinces. Children aged 12–17 years were excluded from the dataset as mental health concerns differ in youth from adulthood, as do experiences of food insecurity in food insecure households [31]. Respondents aged 65 and older were excluded because seniors have the lowest levels of household food insecurity of the adult demographic in Canada, likely related to receiving seniors’ pensions [32]. They also report different mental health problems including more cognitive impairment than working-age adults [33]. In addition, because of challenges of food supply related to isolated geographic areas such as Canada’s Northern Territories [34], only respondents from the 10 provinces were included. 

Provincial participation in the CCHS is dependent on whether modules of the survey were considered core or optional content in each survey cycle; the measurement of household food insecurity via the Household Food Insecurity Survey Module (HFSSM) was optional in the CCHS 3.1; for that cycle, Newfoundland, Labrador, New Brunswick, Manitoba, and Saskatchewan declined participation [35]. In the 2009–2010 cycle, Prince Edward Island and New Brunswick declined participation in the HFSSM [36]. Pooling four cycles and bootstrapping circumvents problems related to generalizability of the results to the ten provinces, given a substantial sample size was still collected in each of the provinces. Given its importance to the research question, only households who provided a response to the HFSSM were included. After applying exclusions, the total sample size was N = 302,683.

### 2.3. Measures

#### 2.3.1. Household Food Security Survey Module 

Household food insecurity in Canada is measured through the HFSSM. This 18-item questionnaire has been internationally validated and translated into many languages [9]. The HFSSM assesses the food security situation of adults as a group and children as a group within the household over the past 12 months. The HFSSM includes 10 questions measuring household food insecurity in adults and 8 questions measuring household food insecurity in children [30]. Typically, Statistics Canada will compile these to create a derived variable measuring three levels of household food security—food secure, moderate food insecurity, and severe food insecurity. For this study, a four-category household food insecurity variable was used, adding marginal food insecurity, which has demonstrated predictive power in increasing risk of chronic conditions in Canadian adults [12,19,20,37,38]. A description of the creation of the four-level household food insecurity variable is available in Appendix A.

#### 2.3.2. Mental Health Outcome Variables

Five common mental health outcomes collected in the CCHS were included in the analysis: depressive thoughts in the past month, anxiety disorders, mood disorders, mental health status, and suicidal thoughts in the past year. All five outcomes were self-reported, because of the nature of the survey, but respondents were asked to only respond affirmatively to the anxiety and mood disorder questions if they had been so diagnosed by a health professional. These mental health conditions were selected based on their high response rates and their relatively high prevalence rates in Canada. The module including some mental health variables was optional content; as a result, two of the outcomes used in this study (suicidal thoughts, depressive thoughts) were not asked in all provinces. A detailed description of the five mental health outcome variables is presented in Appendix B.

#### 2.3.3. Demographic and Socioeconomic Covariates

Six demographic variables (age, sex, household composition, homeownership, and highest education level in household) were included as covariates and were assessed for effect modification or confounding on the relationship between household food insecurity and adverse mental health outcomes. In addition, variables that measure respondents’ race (White, Asian, Indigenous, Other), immigration status (immigrated less than 10 years ago, immigrated 10 or more years ago, Canadian-born), main income source (wages, government assistance, other sources), and inflation-adjusted household income (low income, medium-high income) were also included in the analysis. These covariates were included because of their known association with increased levels of household food insecurity [12,16,17,20,39]. Referent groups were selected based on the lowest prevalence of household food insecurity.

Finally, a cycle variable (2005, 2007/2008, 2009/2010, 2011/2012) was included to determine whether macro-level economic events, such as the 2008–2009 recession in Canada, modified or confounded the relationship between household food insecurity and adverse mental health outcomes. 

### 2.4. Statistical Analysis

Data analysis was conducted at the Prairie Research Data Centre (RDC) using STATA statistical software (version 14, StataCorp, College Station, TX, USA). All estimates were generated with sample weights and 500 bootstrap replicates to approximate the Canadian population, that is, 18–64 year old individuals living in the ten provinces.

Univariate descriptive analyses of all study variables were followed by crude binary logistic regression analyses to assess the proportion of each mental health outcome by level of household food security and by sex, separately, in Canadian adults. Sex-adjusted binary logistic regression models were generated to assess the relationship between household food insecurity and the five mental health outcomes. Finally, interaction terms were created for sex and household food insecurity, and those interactions were included in the sex-adjusted binary logistic regression models to assess for effect modification with each mental health outcome. Reduced (by the removal of non-significant covariates) binary logistic regression analyses were conducted on sex-stratified datasets (one dataset for each sex) to visualize the sex gap for each level of household food insecurity on the odds of reporting five adverse mental health outcomes compared with those who are food secure.

## 3. Results

Table 1 presents the prevalence and 95% confidence intervals (95% CI) of the study variables. The prevalence of adverse mental health outcomes in this population ranged from 5.3% (5.2%–5.4%) reporting fair/poor mental health to 20.0% (19.6%–20.3%) responding that they had had depressive thoughts in the past month. Approximately 11.8% of the population fulfilled the criteria for some level of household food insecurity (3.7% marginal, 6.7% moderate, and 1.4% severe). Females comprised 50.9% (50.8%–50.9%) of the population.

Table 2 presents results from the crude binary logistic regression analysis. The odds ratios were converted to prevalence, and 95% CI are reported for each mental health outcome by level of household food insecurity and by sex, separately. Table 2 also presents the crude sex gap in the reporting of depressive thoughts in the past month, anxiety disorders, mood disorders, suicidal thoughts in the past year, and fair or poor mental health in this population. Table 2 shows that females have a higher prevalence of reporting four out of five mental health outcomes, prior to adjusting for covariates. Males and females have a statistically significant equal prevalence of reporting having had suicidal thoughts in the past year, prior to adjusting for covariates.

Table 3 presents the sex-adjusted odds of reporting five adverse mental health outcomes for each level of household, compared with those reporting household food security. All five adverse mental health outcomes show increasing odds of reporting adverse mental health outcomes with increasingly severe household food insecurity adjusted for sex, compared with those who are food secure. No interaction, that is, no effect modification, was observed between household food insecurity and sex for any of the five adverse mental health outcomes. Table 3 does, however, show that the sex gap for four of the five mental health conditions persists when controlling for household food insecurity. In sum, household food insecurity at any level is associated with increased odds of reporting five mental health outcomes, compared with those reporting food security, and the sex gap remains when household food insecurity is held constant for all mental health conditions, except suicidal thoughts in the past year. 

**Figure 1:**
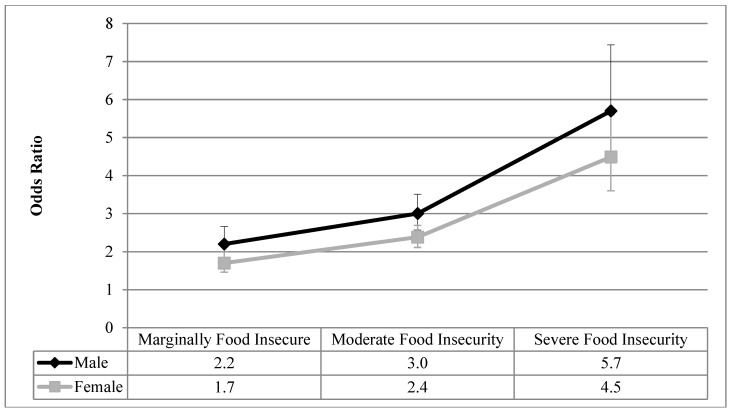
Odds ratio of reporting depressive thoughts in the past month for each level of household food insecurity stratified by sex, compared with food secure; results from reduced binary logistic regression.

**Figure 2:**
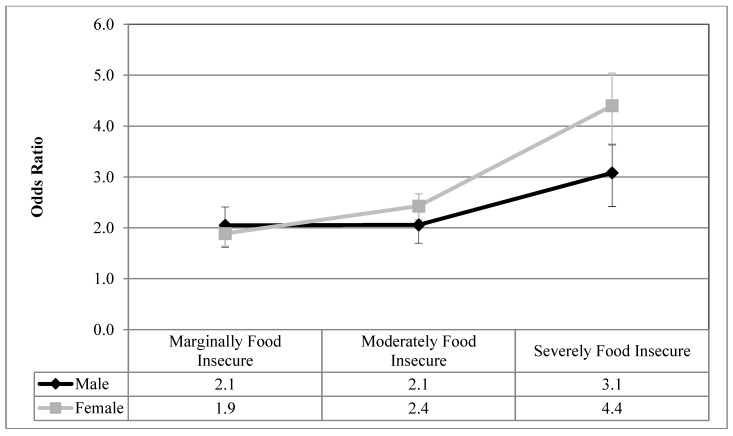
Odds ratio of reporting anxiety disorders for each level of household food insecurity stratified by sex, compared with food secure; results from reduced binary logistic regression.

**Figure 3:**
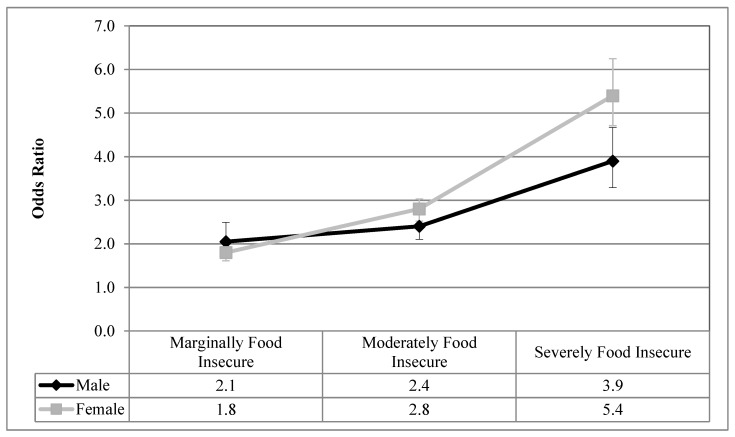
Odds ratio of reporting mood disorders for each level of household food insecurity stratified by sex, compared with food secure; results from reduced binary logistic regression.

**Figure 4:**
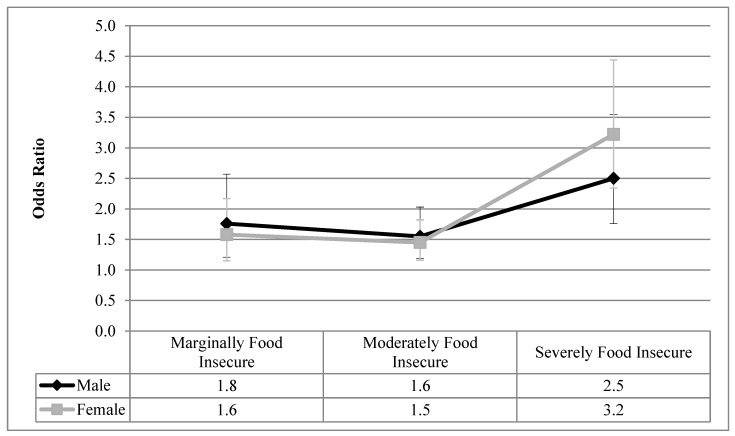
Odds ratio of reporting suicidal thoughts in the past month for each level of household food insecurity stratified by sex, compared with food secure; results from reduced binary logistic regression.

**Figure 5:**
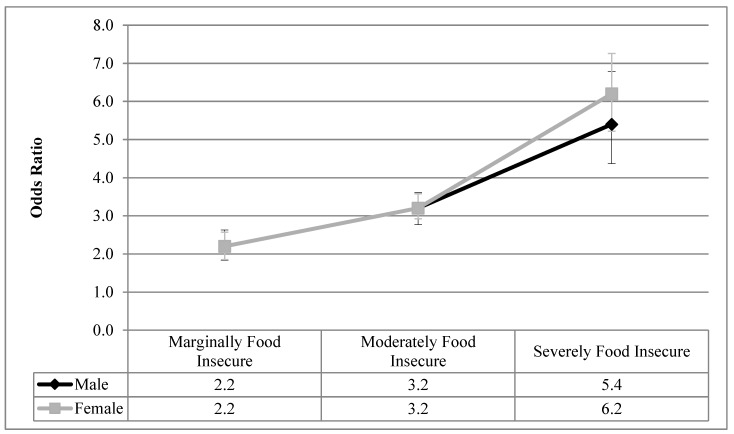
Odds ratio of reporting fair/poor mental health status for each level of household food insecurity stratified by sex, compared with food secure; results from reduced binary logistic regression.

Figure 1, Figure 2, Figure 3, Figure 4 and Figure 5 visualize the results from the reduced binary logistic regression analysis stratified by sex. These figures show, separately for males and females, the adjusted odds ratio of respondents experiencing each level of household food insecurity in turn reporting five mental health outcomes, compared with those reporting that they are food secure. The results, adjusted for significant covariates, show that males and females with any level of household food insecurity have no statistically significant difference in the odds ratio for each mental health outcome, compared to those reporting household food security.

## 4. Discussion

In recent years, mental health researchers have recognized that separate social locations such as sex and race must be considered together, but in much of the scholarship focusing on mental health conditions, these locations are often treated as independent variables [43]. This type of analysis results in a lack of observability of within-group differences among individuals in different social categories. 

From a review of the literature, sex and household food insecurity are two important variables related to the reporting of adverse mental health outcomes. Empirically, this study examined how sex and household food insecurity interact and how that statistical interaction impacts the reporting of mental health outcomes. We first showed that, prior to the inclusion of household food insecurity in the analysis, females reported higher prevalence of depressive thoughts in the past month, anxiety disorders, mood disorders, and fair or poor mental health, compared with males (Table 2), which is aligned with decades of reporting on the sex gap in common mental health outcomes [1,2,3]. Table 3 shows that even after controlling for sex, household food insecurity was associated with high odds ratios of reporting five common mental health outcomes compared with food secure respondents, and these odds ratios increase with the severity of household food insecurity. Upon analyzing the interaction between household food insecurity and sex (Table 3), sex was found to not be an effect modifier on the relationship between household food insecurity and all five adverse mental health outcomes. Therefore, males and females reporting each level of household food insecurity had statistically equal odds ratios of reporting these adverse mental health outcomes, compared with those who are food secure. In order to confirm this finding, sex-stratified reduced binary logistic regression analyses were conducted for each mental health outcome, and the results are presented graphically in Figure 1, Figure 2, Figure 3, Figure 4 and Figure 5. The overlapping confidence intervals at each level of household food insecurity indicate that males and females, at each level of household food insecurity, report statistically similar odds of five common mental health outcomes, compared with food secure respondents. Males and females who experience household food insecurity (a chronically stressful experience) may be equally at risk of reporting mental health problems due to their disadvantaged position. It appears that working age adult males’ lower rates of reporting or succumbing to mental health outcomes [44,45] are reduced to non-significance once household food insecurity is considered.

This is a novel finding and indicates that the often-reported sex gap in mental health may be true among those who are food secure (who represent most Canadian adults), but among a distinctly disadvantaged population (food insecure), males and females appear to experience a similar mental health burden. Another study focused on the interactions between household food insecurity and sex and its relationship with mental health outcomes, in a high-income country. Carter and associates [46] examined the association between a binary food insecurity measure and psychological distress in New Zealand for males and females separately. The authors found that the sex gap was substantially reduced in the stratified model, but that food insecure females had slightly higher odds of psychological distress than males (using a *p*-value of <0.1), compared with those who are food secure [46]. The present study advances this work by examining this relationship using a more precise four-level household food insecurity measure that was generated using an internationally adopted and validated tool (i.e., HFSSM). In addition, the present study examines five common adverse mental health outcomes.

Much of the research on the impact of gender on household food insecurity and mental health has been conducted with a study population comprised of females only [16,17,19]. Our results indicate that the relationship between household food insecurity and five adverse mental health outcomes is equally strong in males. Therefore, the chronic stresses associated with household food insecurity [22] could be narrowing the commonly observed sex gap in mental health. This study’s findings could indicate that the experience of males and females in food insecure households may be similar. Both groups likely experience substantial psychological, physical, and social stresses (e.g., guilt, shame, powerlessness, and inequality) [22,28] as a result of not having enough money to feed themselves or their families. 

Our findings suggest that males and females in a food insecure household experience a similar mental health burden as a result of sharing a more similar social location compared with food secure members of their own sex. The sex gap in mental health outcomes that is often reported for the general population is largely comprised of food secure respondents and appears to have masked the food insecure sub-group’s experience of mental health problems, if this important variable is not considered.

### 4.1. Limitations

The CCHS does not survey some groups that are particularly vulnerable to household food insecurity and mental health problems, specifically First Nations people living on-reserve, homeless populations, and those living in remote regions [47,48]. Researchers estimate that there could be as many as 470,000 additional food insecure people living in Canada that are not included in Statistics Canada’s estimate [49]. We cannot assume that the social location of household food insecurity for men and women in these circumstances is the same as the CCHS sample.

While it would be preferable to speak only in terms of gender, the CCHS only delineates by sex and, therefore, sex is used as a variable of interest in this study. Given the self-reported nature of the CCHS, there is potential for measurement error in the mental health outcomes, particularly as a result of social desirability bias—this would result in an underestimation of mental health burden.

The CCHS uses two methods of data collection—computer assisted telephone interviewing (CATI) and computer-assisted person interviewing (CAPI). While there is some evidence in the literature of statistically equal prevalence of self-reported mental health status using CATI and CAPI [50], face-to-face interviewing yields slightly higher reporting of household food insecurity than CATI [51]. This may result in an underestimation of household food insecurity for those responding using CATI.

Finally, type 1 error (the probability of rejecting a null hypothesis that is in fact true [52]) is a threat with a large sample size. In order to circumvent this problem, a bootstrapping method was employed, which effectively narrowed the confidence intervals to increase the difficulty of rejecting the null hypothesis.

### 4.2. Strengths

This study employs a large and robust dataset, with enough power to examine a four-level household food insecurity variable, using the internationally validated HFSSM. This study uses a nationally representative survey that results in generalizable findings to 98% of the Canadian population aged 18–64 living in the ten provinces. The use of a four-level household food insecurity variable is rare in the literature, despite the predictive power of the marginal group [12,19,37,38]. 

Finally, this study is novel in that it is one of only two studies to examine the interaction between sex, household food insecurity, and mental health outcomes in high-income countries [46], and the first to examine this phenomenon in Canada, with a four-level food insecurity variable.

## 5. Conclusions

Although the sex gap in mental health outcomes has been observed and re-examined for decades, few studies have considered whether these important social determinants, sex and food insecurity, have a multiplicative effect on the odds of reporting of mental health problems. 

This study showed that the well-documented sex gap in mental health outcomes was reduced to non-significance when household food insecurity was reported. Therefore, household food insecurity appears to act as a chronically stressful condition that overwhelms the capacity of males to either withstand reporting mental health conditions or actually succumb to them. This study suggests that household food insecurity is a social location with public health implications. The high odds of reporting adverse mental health outcomes seen among males experiencing household food insecurity, compared with food secure males, suggest that there is a distinct mental health burden among males experiencing household food insecurity, and that this previously overlooked group is deserving of further study. 

Finally, household food insecurity is a modifiable stressor within the complex interplay of sex and mental health. Given the lack of a sex gap in mental health among those with household food insecurity, addressing food insecurity with progressive policy change could result in mental health gains for women, as well as men who share this vulnerability.

## Figures and Tables

**Table 1:** Prevalence (%) and 95% confidence intervals (CI) of study variables (N = 302,683). CCHS—Canadian Community Health Survey.

**Variable**	**Categories**	**Percent**	**95% CI**
**Outcome**			
Depressive Thoughts in the Past Month	Yes	20.0	19.6–20.3
Anxiety Disorders	Yes	5.8	5.7–6.0
Mood Disorders	Yes	7.2	7.0–7.3
Suicidal Thoughts in the Past Year	Yes	19.7	18.7–20.7
Mental Health Status	Fair/Poor	5.3	5.2–5.4
**Exposure**			
Household Food Insecurity Level	Food Secure	88.2	88.0–88.4
	Marginal Food Insecurity	3.7	3.5–3.8
	Moderate Food Insecurity	6.7	6.5–6.9
	Severe Food Insecurity	1.4	1.3–1.5
**Covariate**	**Categories**	**Mean**	**Standard Deviation**
Age	Continuous (18–64)	42.8	13.5
**Covariate**	**Categories**	**Percent**	**95% CI**
Sex	Male	49.1	49.1–49.2
	Female	50.9	50.8–50.9
Household Composition	Unattached, living alone	12.5	12.3–12.7
	Single living with others	5.1	5.0–5.3
	Couple, no kids	25.3	25.0–25.5
	Couple with kids <25	45.0	44.7–45.3
	Lone parent, kids <25	6.1	5.9–6.3
	Other/multi-family	6.0	5.9–6.2
Marital Status	Common-law or Married	65.2	64.9–65.4
	Divorced, Widowed, or Separated	9.2	9.0–9.4
	Single	25.7	25.4–25.9
Inflation-Adjusted Income ^a^	Low	5.8	5.6–5.9
	Med-High	94.2	94.1–94.4
Income Source	Wages & Salary	88.9	88.7–89.1
	Social Assistance ^b^	9.3	9.2–9.5
	Other ^c^	2.7	2.6–2.8
Race	White	79.2	78.9–79.6
	Asian	11.7	11.4–11.9
	Indigenous	2.6	2.5–2.7
	Other ^d^	6.5	6.3–6.7
Education	Post-Secondary Degree	80.5	80.2–80.7
	Some Post-Secondary	5.4	5.2–5.5
	High School Grad	9.8	9.7–10.0
	Less than High School	4.4	4.2–4.5
Immigration	Immigrated ≥10 years	15.7	15.5–16.0
	Immigrated <10 years	7.5	7.3–7.7
	Canadian Born	76.7	76.4–77.0
Homeownership	Homeowner	73.5	73.1–73.8
	Renter	26.5	26.2–26.9
Cycle of CCHS	3.1	22.2	22.1–22.3
	2007/2008	25.5	25.4–25.6
	2009/2010	25.6	25.6–25.7
	2011/2012	26.6	26.6–26.7

^a^ Derived from respondent’s total household income before taxes adjusted by Canadian inflation rates for the year the respondent was surveyed [40]. Inflation adjusted income was ranked (low-lower middle, middle, upper middle, and highest) based on the number of people in household and national income thresholds [41,42]. The four-level variable was dichotomized into low and medium-high income. ^b^ includes the following: benefits from Canada or Quebec pension plan, old age security and guaranteed income supplement, provincial or municipal social assistance or welfare, and child tax benefit. ^c^ includes the following: retirement pensions, child support, alimony, employment insurance, worker’s compensation board, and other. ^d^ includes those who identify as Black, Latin American, Arab, and Other (multi-racial).

**Table 2:** Results from crude binary logistic regression of household food insecurity and by sex, separately, on five adverse mental health outcomes, presented as prevalence (%) and 95% confidence intervals.

Variable Category	Depressive Thoughts in the Past Month	Anxiety Disorders	Mood Disorders	Suicidal Thoughts in the Past Year	Fair/Poor Mental Health
Household Food Insecurity Level					
Food Secure	17.5 (17.2, 17.9)	4.8 (4.7, 4.9)	5.8 (5.7, 5.9)	16.8 (15.7, 17.8)	4.0 (3.9, 4.1)
Marginally Food Insecurity	31.1 (28.7, 33.5)	9.9 (9.1, 10.8)	11.4 (10.5, 12.2)	25.6 (21.2, 30.0)	9.2 (8.3, 10.1)
Moderately Food Insecurity	39.8 (37.8,4 1.7)	13.6 (12.8, 14.3)	17.4 (16.6, 18.3)	24.8 (22.0, 27.7)	15.0 (14.2, 15.8)
Severely Food Insecurity	59.3 (55.2, 63.4)	25.4 (23.5, 27.3)	34.2 (32.0, 36.4)	41.0 (36.1, 45.9)	31.1 (28.9, 33.4)
Sex					
Male	15.1 (14.6, 15.7)	4.1 (4.0, 4.3)	5.0 (4.8, 5.2)	20.9 (19.4, 22.4)	4.8 (4.6, 5.0)
Female	24.7 (24.1, 25.2)	7.5 (7.3, 7.7)	9.3 (9.1, 9.5)	18.8 (17.5, 20.1)	5.8 (5.6, 6.0)

**Table 3:** Sex-adjusted binary logistic regression models of household food insecurity and five adverse mental health outcomes, including food insecurity and sex interactions.

Variable Category	Model 1: Depressive Thoughts in the Past Month	Model 2: Anxiety Disorders	Model 3: Mood Disorders	Model 4: Suicidal Thoughts in the Past Year	Model 5: Fair/Poor Mental Health
**Odds Ratio (95% Confidence Interval)**					
**Household Food Insecurity Level (food secure = ref)**					
Marginal Food Insecurity	2.3 ** (2.0, 2.8)	2.2 ** (1.9, 2.7)	2.3 ** (1.9, 2.7)	1.8 * (1.3, 2.6)	2.4 ** (2.1, 2.9)
Moderate Food Insecurity	3.2 ** (2.8, 3.7)	2.8 ** (2.5, 3.2)	3.2 ** (2.9, 3.6)	1.8 ** (1.4, 2.4)	4.3 ** (3.8, 4.8)
Severe Food Insecurity	8.2 ** (6.3, 10.6)	6.3 ** (5.4, 7.3)	8.4 ** (7.2, 9.7)	3.0 ** (2.2, 4.1)	11.0 ** (9.2, 13.1)
**Sex (male = ref)**					
Female	1.9 ** (1.8, 2.0)	1.8 ** (1.7, 1.9)	1.9 ** (1.8, 2.0)	0.9 * (0.7, 1.0)	1.2 ** (1.1, 1.2)
Marginal * Female	0.8 (0.7, 1.0)	0.9 (0.8, 1.2)	0.9 (0.7, 1.0)	0.9 (0.6, 1.5)	1.0 (0.8, 1.2)
Moderate * Female	0.9 (0.7, 1.1)	1.1 (0.9, 1.3)	1.0 (0.9, 1.2)	0.9 (0.6, 1.2)	1.0 (0.8, 1.1)
Severe * Female	0.7 (0.5, 1.0)	1.2 (0.9, 1.4)	1.0 (0.9, 1.3)	1.3 (0.9, 2.0)	1.0 (0.8, 1.2)

* *p* < 0.05, ** *p* < 0.001.

## Appendix A

**Table A1:** Description of Household Food Insecurity Variable Derived from Household Food Security Survey Module.

Level of Household Food Insecurity	Description of Level	Number of Affirmative Responses
Food Secure	No financial constraints on the ability to fill household’s food need.	0 to adult or child food situation questions
Marginally Food Insecure	Worry about running out of food due to financial constraints.	1 food situation question
Moderately Food Insecure	Reductions in quality and/or quantity of food due to financial constraints.	2–5 adult food situation questions or 2–4 child food situation questions
Severely Food Insecurity	Reductions in food intake, missing meals and at its most extreme going a full day without food.	6+ adult food situation questions or 5+ child food situation questions

* adapted from [8].

## Appendix B

**Table A2:** Description of Outcome Variables Included in Study.

Name of Variable	Level of Measurement	Survey Question	Description
Depressive Thoughts in the Past Month	Binary (Yes, No)	“During the past month, about how often did you feel sad or depressed?”	Those who responded all of the time, most of the time, some of the time were coded into the “yes” group. All other respondents were coded into the “no” group.
Major Depressive Episodes in the Past Year	Binary (Yes, No)	The Composite International Diagnostic Interview Short Form (CIDI-SF) measures Major Depressive Episodes (MDE). This subset of questions assesses the depressive symptoms of respondents who felt depressed or lost interest in things for 2 weeks or more in the last 12 months. Respondents are screened into the CIDI-SF based on affirmative responses to the following 2 screening questions, if a respondent answers affirmatively to the screening questions, their depression level is measured based on 7 additional questions.	The classification of depression is based on an affirmative response to the original screening question and 5 out of 9 of the depression questions. This corresponds to a 90% predictive probability of caseness, which closely aligns with the DSM-5 diagnostic guidelines for MDE in adults [53]. This probability expresses the chance that the respondent would have been diagnosed as having experienced a Major Depressive Episode in the past 12 months had they completed the CIDI Long-Form [30].
Anxiety Disorder	Binary (Yes, No)	“Do you have an anxiety disorder such as phobia, obsessive-compulsive disorder or a panic disorder?”	Respondents are reminded that the question is only referring to those conditions diagnosed by a health professional.
Mood Disorder	Binary (Yes, No)	“Do you have a mood disorder such as depression, bipolar disorder, mania or dysthymia?”	Respondents are reminded that the question only refers to those conditions diagnosed by a health professional.
Suicidal Thoughts in the Past Month	Binary (Yes, No)	“Have you ever seriously considered committing suicide or taking your own life? Has this happened in the past 12 months?”	This variable was recoded into a dichotomous variable. In addition, those who answered “not applicable: were coded into the “no” group, given they answered negatively to this question in an earlier prompt.
Self-Reported Mental Health Status	Binary (Fair/Poor, Good/Very Good/Excellent)	“In general, would you say your mental health is: excellent, very good, good, fair, or poor?”	This variable was recoded into a dichotomous variable. “Fair/poor” or “Good/very good/excellent”. This variable has been validated and is a reliable measure of general mental health [54].
